# Bilateral Piriform sinus fistulas: a case study and review of management options

**DOI:** 10.1186/s40463-018-0258-y

**Published:** 2018-02-14

**Authors:** Deanna Lammers, Ross Campbell, Jorge Davila, Johnna MacCormick

**Affiliations:** 1 University of Ottawa Faculty of Medicine, Roger Guindon Hall, 451 Smyth Rd., Ottawa, ON K1H 8M5 Canada; 2 Children’s Hospital of Eastern Ontario, 401 Smyth Road, Ottawa, ON K1H 8L1 Canada

**Keywords:** Piriform sinus fistula, Fourth Branchial fistula, Third Branchial fistula, Branchial arch abnormality, Suppurative Thyroiditis, Endoscopic repair

## Abstract

**Background:**

Piriform sinus fistulas occur due to developmental abnormalities of the third and fourth branchial arches, and almost always occur unilaterally. They generally present as recurrent abscesses in the anterior-inferior neck, with concurrent thyroiditis. They have conventionally been managed with complete removal of the sinus tract, and thyroidectomy if required; however, endoscopic approaches have been increasingly favored. Herein we describe a case of bilateral piriform sinus fistulas, and present a review of the literature concerning their endoscopic management.

**Case presentation:**

Our patient was determined to have bilateral piriform sinus fistulas based on computer tomography, magnetic resonance imaging and microlaryngoscopy. We performed electrocauterization of the proximal fistula tracts, followed by injection of fibrin sealent. Our patient has not had a recurrence in the ten months since his procedure. There were no complications.

Twenty-three articles describing an endoscopic approach to these fistulas were identified through PubMed, and a search through the references of related articles was completed.

**Conclusion:**

Of one hundred and ninety-five patient cases we reviewed, an endoscopic procedure success rate of 82% and complication rate of 5.6% was determined. Piriform sinus fistulas that occur bilaterally are a rare congenital abnormality of the neck. Endoscopic approaches are an acceptable alternative option to open procedures, with similar success and a lower rate of complications.

## Background

Third and fourth branchial apparatus anomalies, commonly referred to as piriform sinus fistulas (PSFs), are sinus tracts and fistulas that develop from the piriform sinus. They occur more commonly on the left side, and typically present in childhood with recurrent acute suppurative thyroiditis and neck abscess often following an upper respiratory tract infection [1–3]. Infants and neonates may have respiratory distress, stridor, dysphagia and feeding difficulties due to tracheal compression from the abscess [3, 4]. Thyroid function is usually normal [1].

PSFs are uncommon and can be difficult to diagnose, due in part to their non-specific presentations [1]. Furthermore, bilateral PSFs are extremely rare, with a thorough literature search revealing only four other patients described with this condition [5–7]. Herein, we present a case of a patient with bilateral piriform sinus fistulas, and review their management.

## Case presentation

**Fig. 1:**
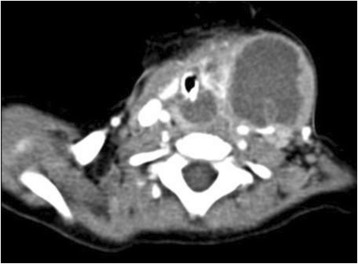
Axial enhanced CT showing involvement of the thyroid gland with surrounding multiloculated abscess

Our patient initially presented at 10 months of age with rapid development of a mass in the left neck that was tender, firm and erythematous. It was associated with fever as well as dysphagia, and decreased oral intake. The patient had no significant past medical history, nor family history. A lateral neck x-ray suggested a retropharyngeal infectious process. After 48 h of antibiotic treatment, there was minimal clinical improvement; a computer tomography (CT) scan of the neck demonstrated a 5.4 × 3.5 × 4.2 cm left neck abscess extending to left parapharyngeal and retropharyngeal spaces (Fig. 1). Following imaging, the patient underwent incision and drainage. The wound culture grew Streptococcus viridans and Hemophilus parainfluenzae.

For 6 months after the surgery, the wound persisted to drain intermittently, culminating in a submandibular cellulitis. A CT neck and wound culture at that time were unremarkable, and the patient improved after 5 days of IV antibiotics.

**Fig. 2:**
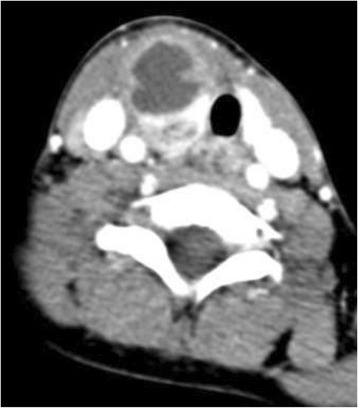
Axial enhanced CT illustrating involvement of the abscess with the right lobe of the thyroid gland

At 8 years of age, the patient again presented with a 4 day history of sore throat, fever, drooling, and neck stiffness and swelling; IV antibiotic therapy was initiated. A CT neck confirmed a bilobed right-sided retropharyngeal abscess measuring 2.3 × 2.9 × 3.3 cm and 2.0 × 2.1 × 2.8 cm in the superior and anterior lobes, respectively. The thyroid gland was noted to be intimately involved with this inflammatory process (Fig. 2). The patient was brought to the operating room for incision and drainage of the abscess. The wound culture was positive for Streptococcus anginosis.

**Fig. 3:**
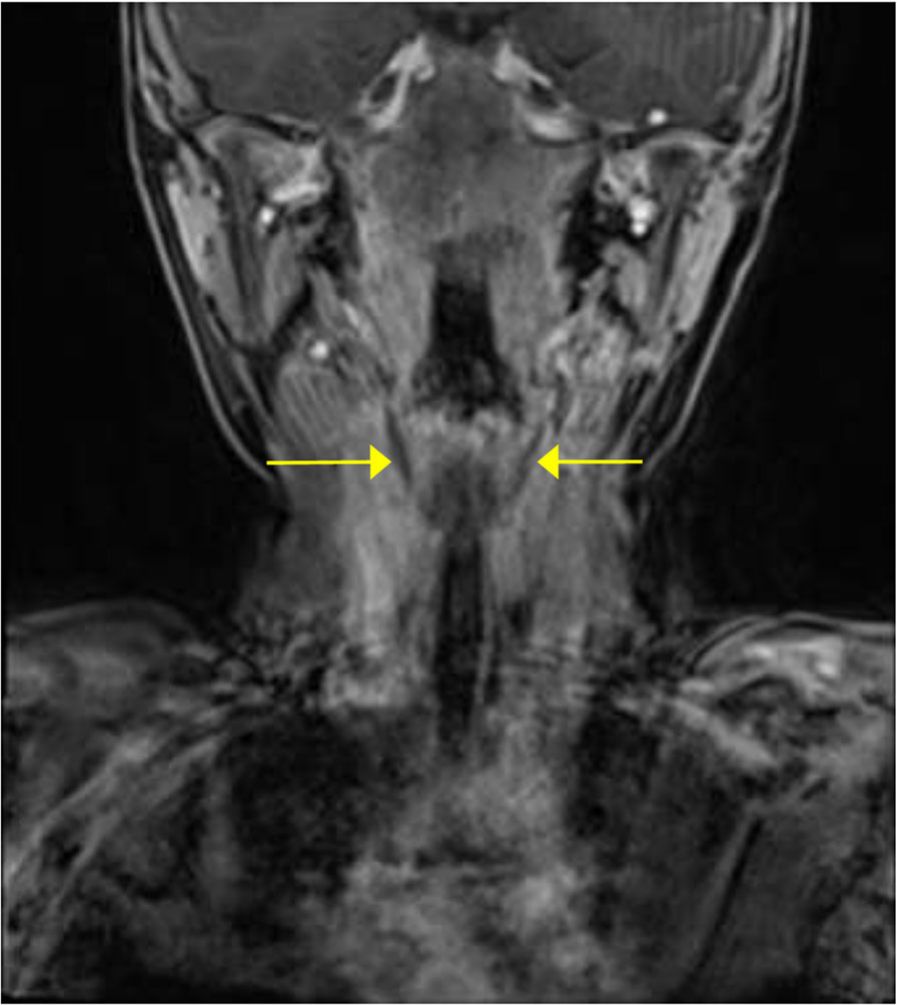
Coronal MRI identifying bilateral tracts from the piriform sinus to the thyroid gland

**Fig. 4:**
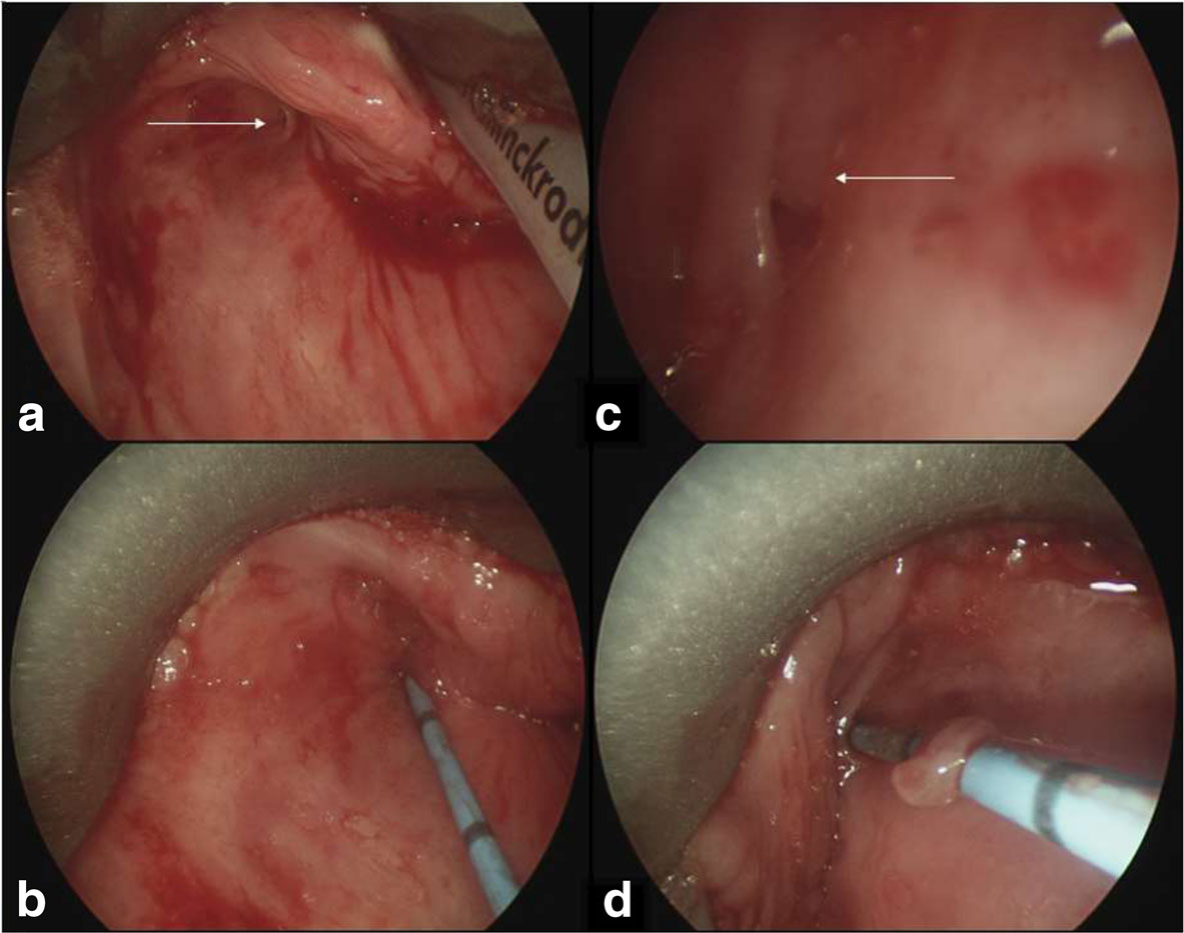
Left piriform sinus fistula seen in (**a**); cannulated in (**b**); Right piriform sinus fistula seen in (**c**); cannulated in (**d**)

Shortly following the resolution of the infection, magnetic resonance imaging (MRI) was performed identifying bilateral communications between the piriform sinuses and thyroid lobes (Fig. 3). Direct laryngoscopy confirmed the diagnosis of bilateral piriform sinus fistula with the passage of 4 Fr ureteric catheters though tracts originating in the piriform sinuses, on both the left and right sides (Fig. 4).

**Fig. 5:**
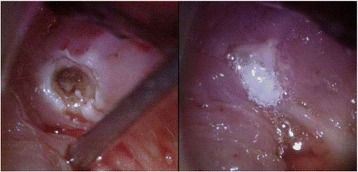
Right piriform sinus fistula following electrocauterization (left) and fibrin sealant into the fistula tract (right)

After lengthy discussions and deliberation and review of the relevant literature, the family opted for endoscopic electrocauterization with fibrin sealant management (Fig. 5). We favoured this approach as the PSFs were bilateral, and therefore a total thyroidectomy may have been required for definitive surgical management.

Cauterization and obliteration of the bilateral PSFs was performed without complication. First, a flexible catheter was passed into the piriform sinus to confirm its location. With the location confirmed, a Bugbee catheter was inserted into the fistula and the edges were cauterized at a setting of 8 W. Once adequate cauterization was achieved, Tisseel was injected into the fistula tract. This procedure was performed in both the left and right PSFs. A follow-up 6 months with direct laryngoscopy confirmed closure of both tracts. There has been no recurrence 10 months following the procedure, at the time of writing of this manuscript.

## Discussion and conclusions

A thorough literature search about the endoscopic management for piriform sinus fistulas was performed. To identify journal articles, a literature search using PubMed was conducted using the filters English language and years 1998-2017. The references in each article were also reviewed to find additional papers. Twenty-three articles were identified as relevant studies. Table 1 lists the year the study was published, number of patients enrolled, patient characteristics (age, gender), techniques used, success rate, amount of follow-up and complication rate. A procedure was considered a success if did not have to be repeated for either recurrence, or incomplete fistula tract closure. For research groups that had multiple publications using repeat patient cases, only their last publication was included.

**Table 1:** Endoscopic repair patient demographics, techniques used, success rates, follow-up and complication rates

Author	Year	Number of Patients	Patient Age	Patient Gender	Technique	Success rate	Duration of follow-up	Complication rate
Shrime [29]	2003	1	1d	F	CC with silver nitrate	100%	–	100% (Transient vocal cord palsy)
Cigliano [12]	2004	1	9	F	FS repeated at short term interval three times	100%	15 m	0%
Ahmed [14]	2008	3	3-9y	1 M 2F	Secondary EC following failed surgical excision	100%	9-13 m	0%
Pereira [8]	2008	2	2-18y	2 M	CC with silver nitrate	100%	2y	0%
Chen [13]	2009	9	3-16y	1 M 8F	EC +/− polyglactin sutures	78%	7 m-8y	0%
Miyauchi [30]	2009	12	14-31y	2 M 10F	CC with 30% TCA	83%	4-21 m	0%
Leboulanger [15]	2010	19	1d-18y	–	2 EC 13 CO_2_ Laser 4 Thulium laser	68%	6 m-5y	0%
Bajaj [16]	2011	3	<1y	–	EC	100%	6w	0%
Zhang [21]	2012	1	15y	M	CC	0%	5y	0%
Cha [9]	2013	44	–	–	31 CC with 20-40% TCA 13 Secondary CC with TCA following failed surgical excision	77%	18 m-18y	0%
Park [20]	2013	2	13 m-5y	1 M 1F	CC with 30% TCA	100%	7-18 m	100% (Transient vocal cord palsy)
Watson [17]	2013	5	2-12y	1 M 4F	1 EC 2 CO_2_ Laser 2 CC with silver nitrate	100%	11-41 m	0%
Parida [19]	2014	3	11-12y	1 M 2F	2 CC with silver nitrate 1 Secondary CC with silver nitrate following failed surgical excision	100%	2-3y	0%
Sun [11]	2014	22	6 m-14y	7 M 15F	EC	91%	1 m-14y	0%
Wong [28]	2014	2	10-14y	1 M 1F	1 EC 1 Secondary EC following failed surgical excision	50%	4y	50% (mild hoarseness that resolved within 2 weeks)
Hwang [24]	2015	13	1.5-15y	9 M 4F	CC with 20% TCA	54%	5.5y (median)	0%
Josephson [1]	2015	1	7y	F	CO2 laser with chromic suture	100%	4y	0%
Kamide [18]	2015	1	20y	F	Electrocauterization	100%	1y	0%
Abbas [25]	2016	1	12y	F	Electrocauterization	100%	22 m	0%
Di Nardo [22]	2016	1	3y	F	Secondary Glubran 2 sealing following 4 failed surgical excisions	100%	6y	0%
Huang [27]	2016	5	5-7y	3 M 2F	KTP laser assisted EC with FS	80%	7-36 m	0%
Matsuzaki [26]	2016	2	9-26y	1 M 1F	Endoscopic partial resection with polydioxanone suture	100%	1-2y	0%
Zhang [23]	2016	42	–	–	11 EC 31 Coblation cauterization	88%	2-40 m	7% (temporary hoarseness)

*EC* electrocauterization, *CC* Chemocauterization, *FS* fibrin sealent, *TCA* trichloroacetic acid, *M* male, *F* female, *m* month, *y* years

A PubMed search was also used to find cases of bilateral piriform sinus fistulae. No filters were used. Four cases were found from three publications.

From one hundred and ninety-five cases of piriform sinus fistulas, based on 23 studies, we calculated an endoscopic success rate average of 82% and complication rate of 5.6%. Post-operative follow-up times ranged from 6 weeks to 18 years with an average of 35.5 months. Patient ages ranged from newborns to 31 years, and there was a gender distribution of 1.8 females to 1 male (56 females, 31 males). However, the gender and ages of the patients in the studies could not be identified for 55% of patient cases presented. All the studies were published from 2003 to 2016.

PSFs are notoriously difficult to diagnose. While neck abscesses are a common entity in pediatric otolaryngology patients, PSFs are rare. With a repeated history of anterior neck abscesses, or when imaging identifies thyroid gland involvement, this diagnosis should be considered. Confirmation is typically done with direct laryngoscopy, which has a positive predictive value (PPV) of 90%, or barium swallow (PPV 88%) [1, 2]. Management of a piriform sinus fistula involves antibiotics and incision and drainage for acute infections, and observation, followed by surgery or endoscopic techniques for long-term management.

In the acute setting, the choice of antibiotic should reflect typical oral flora as well as *Staphylococcus aureus* [2]. Surgical drainage for source control is indicated where there is abscess formation [1].

Considering long-term management, observation may be considered, especially for asymptomatic PSFs; however, 89-94% of patients will continue to have recurrent infections [2, 3]. Therefore, further treatment is usually necessary.

Surgical removal of the sinus tract, and the thyroid gland if required, is typically curative, and has been the standard of care for many years [1, 9]. However, excising the entire fistula tract can be very technically challenging, especially if the patient has significant scarring from repeated infections and prior surgical drainages. Incomplete excision may lead to recurrence [1]. In addition, the recurrent laryngeal nerve is at risk for injury leading to potential vocal cord paralysis. Salivary fistulas, hemorrhage, wound infection, cicatrises, Horner syndrome and injury to branches of the facial nerve are other possible complications. Reported success rates range from 85 to 100% [2–4, 6] with a complication rate of 5-6% [2, 3].

An alternative to surgery is endoscopic electrocautery or chemocauterization, followed by fibrin sealing or endoscopic suture ligation if necessary. This closes off the proximal portion of the tract, preventing leakage of pharyngeal contents into the sinus. Endoscopic techniques have recently been shown to be safe and effective [9, 10], and can be performed as an outpatient procedure, thereby reducing hospitalization and associated costs. The associated risks are lower compared to an external technique, also with the advantage of lacking a surgical incision and consequent scar [9, 11]. However, this is a newer procedure, and data regarding long-term results is scarce. Recent studies suggest the success rate is equivalent to, or slightly less than surgery, with success rate average of 82% (Table 1) [1–3, 8–29].

In summary, piriform sinus fistulas are uncommon developmental anomalies in children. The presentation of repeated anterior neck abscesses, particularly if the thyroid gland is involved should prompt the clinician to consider this entity, and trigger an appropriate work-up. As the fistulas may occur bilaterally, a careful inspection of both sides is required during laryngoscopy. Definitive management has classically been performed through an excision of the tract. Endoscopic electrocautery of the tract may be an acceptable alternative, with comparable success and a lower rate of complications.

## Abbreviations

CT: Computer tomography
MRI: Magnetic resonance imaging
PPV: Positive predictive value
PSF: Piriform sinus fistula

## References

[1] Josephson GD, Black K. A review over the past 15 years of the management of the internal piriform apex sinus tract of a branchial pouch anomaly and case description. Ann Otol Rhinol Laryngol. 2015;124:947–52. 10.1177/0003489415593554 26215722

[2] Nicoucar K, Giger R, Pope HG, Jaecklin T, Dulguerov P (2009). Management of congenital fourth branchial arch anomalies: a review and analysis of published cases. J Ped Surg.

[3] Nicoucar K, Giger R, Jaecklin T, Pope HG, Dulguerov P. Management of congenital third branchial arch anomalies: a systematic review. Otolaryngol Head Neck Surg. 2010; 10.1016/j.otohns.2009.09.001. Accessed 16 May 2016. 10.1016/j.otohns.2009.09.001 20096218

[4] Liberman M, Kay S, Emil S, Flageole H, Nguyen LT, Tewfik TL, et al. Ten years of experience with third and fourth branchial remnants. J Pediatr Surg. 2002; 10.1053/jpsu.2002.32253. Accessed 16 May 2016. 10.1053/jpsu.2002.32253 11987078

[5] Rossiter JL, Topf P (1991). Acute suppurative thryoiditis with bilateral piriform sinus fistulae. Otolaryngol Head Neck Surg..

[6] Xiao X, Zheng S, Zheng J, Zhu L, Dong K, Shen C, et al. Endoscopic-assisted surgery for pyriform sinus fistula in children: experience of 165 cases from a single institution. J Pediatr Surg. 2014; 10.1016/j.jpedsurg.2013.11.004. Accessed 16 May 2016. 10.1016/j.jpedsurg.2013.11.004 24726124

[7] Dean RL, Donovan T (2006). Bilateral pyriform sinus fistulas presenting as recurrent suppurative thyroiditis. Otolaryngol Head Neck Surg.

[8] Pereira KD, Smith SL. Endoscopic chemical cautery of piriform sinus tracts: a safe new technique. Int J Pediatr Otorhinolaryngol. 2008; 10.1016/j.ijporl.2007.10.007. Accessed 16 May 2016. 10.1016/j.ijporl.2007.10.007 18031833

[9] Cha W, Cho SW, Hah JH, Kwon TK, Sung MW, Kim KH (2013). Chemocauterization of the internal opening with trichloroacetic acid as first-line treatment for pyriform sinus fistula. Head Neck.

[10] Lachance S, Chadha NK. Systematic review of endoscopic obliteration techniques for managing congenital Piriform Fossa Sinus tracts in children. Otolaryngol Head Neck Surg. 2016;154(2):241–46. 10.1177/0194599815613286 26527612

[11] Sun JY, Berg EE, McClay JE (2014). Endoscopic cauterization of congenital Pyriform Fossa Sinus tracts: an 18-year experience. JAMA Otolaryngol Head Neck Surg.

[12] Cigliano B, Cipolletta L, Baltogiannis N, Esposito C, Settimi A (2004). Endoscopic fibrin sealing of congenital pyriform sinus fistula. Surg Endosc Other Interv Tech.

[13] Chen EY, Inglis AF, Ou H, Perkins JA, Sie KCY, Chiara J (2009). Endoscopic electrocauterization of pyriform fossa sinus tracts as definitive treatment. Int J Pediatr Otorhinolaryngol.

[14] Ahmed J, De S, Hore IDB, Bailey CM, Hartley BEJ (2008). Treatment of piriform fossa sinuses with monopolar diathermy. J Laryngol Otol.

[15] Leboulanger N, Ruellan K, Nevoux J, Pezzettigotta S, Denoyelle F, Roger G (2010). Neonatal vs delayed-onset fourth branchial pouch anomalies: therapeutic implications. Arch Otolaryngol Head Neck Surg.

[16] Bajaj Y, Ifeacho S, Tweedie CG, Jephson DM, Albert LA, Cochrane ME, et al. Branchial anomalies in children. Int J Pediatr Otorhinolaryngol. 2011; 10.1016/j.ijporl.2011.05.008. Accessed 16 May 2016. 10.1016/j.ijporl.2011.05.008 21680029

[17] Watson GJ, Nichani JR, Rothera MP, Bruce IA. Case series: endoscopic management of fourth branchial arch anomalies. Int J Pediatr Otorhinolaryngol. 2013; 10.1016/j.ijporl.2013.02.007. Accessed 16 May 2016. 10.1016/j.ijporl.2013.02.007 23478017

[18] Kamide D, Tomifuji M, Maeda M, Utsunomiya K, Yamashita T, Araki K (2015). Minimally invasive surgery for pyriform sinus fistula by transoral videolaryngoscopic surgery. Am J Otolaryngol.

[19] Parida PK, Gopalakrishnan S, Saxena SK. Pediatric recurrent acute suppurative thyroiditis of third branchial arch origin-our experience in 17 cases. Int J Pediatr Otorhinolaryngol. 2014; 10.1016/j.ijporl.2014.08.034. Accessed 16 May 2016. 10.1016/j.ijporl.2014.08.034 25219934

[20] Park JH, Jung YH, Sung MW, Kim KH. Temporary vocal fold immobility after chemocauterization of the pyriform sinus fistula opening with trichloroacetic acid. Laryngoscope. 2013;123:410–3. 10.1002/lary.23530 22847863

[21] Zhang J, Huang S, Li H, Li Y, Chen H, Gu L (2012). Relapsing suppurative neck abscess after chemocauterization of pyriform sinus fistula. Clin Imaging.

[22] Di Nardo G, Valentini V, Angeletti D, Frediani S, Iannella G, Cozzi D, et al. Recurrent pyriform sinus fistula successfully treated by endoscopic Glubran 2 sealing: a rare case and literature review. SAGE Open Med Case Rep. 2016;4:1–4. 10.1177/2050313X16672151 PMC5066581 27781098

[23] Zhang P, Tian X (2016). Recurrent neck lesions secondary to pyriform sinus fistula. Eur Arch Otorhinolaryngol.

[24] Hwang J, Kim SC, Kim DY, Namgoong JM, Nam SY, Roh JL. Excision versus trichloroacetic acid (TCA) chemocauterization for branchial sinus of the pyriform fossa. J Pediatr Surg. 2015;50:1947–53. 10.1016/j.jpedsurg.2015.07.006 26282101

[25] Abbas PI, Roehm CE, Friedman EM, Athanassaki I, Kim ES, Brandt ML (2016). Successful endoscopic ablation of a pyriform sinus fistula in a child: case report and literature review. Pediatric Surg Int.

[26] Matsuzaki H, Makiyama K, Suzuki H, Ohshima T. Prevention of neck infection by endoscopic suture closure of pyriform sinus fistulae: a report of two cases. Braz J Otorhinolaryngol. 2016; 10.1016/j.bjorl.2015.11.012. Accessed 7 Jan 2017. 10.1016/j.bjorl.2015.11.012 PMC9449173 26944366

[27] Huang YC, Peng SSF, Hsu WC (2016). KTP laser assisted endoscopic tissue fibrin glue biocauterization for congenital pyriform sinus fistula in children. Int J Pediatr Otorhinolaryngol.

[28] Wong PY, Moore A, Daya H (2014). Management of third branchial pouch anomalies – an evolution of a minimally invasive technique. Int J Pediatr Otorhinolaryngol.

[29] Shrime M, Kacker A, Bent J, Ward RF (2003). Fourth branchial complex anomalies: a case series. Int J Pediatr Otorhinolaryngol.

[30] Miyauchi A, Inoue H, Tomoda C, Amino N. Evaluation of chemocauterization treatment for obliteration of pyriform sinus fistula as a route of infection causing acute suppurative thyroiditis. 2009; 10.1089/thy.2009.0015. Accessed 8 Jan 2017. 10.1089/thy.2009.0015 19508119

